# Supplementary material for: The interactome of intact mitochondria by cross-linking mass spectrometry provides evidence for coexisting respiratory supercomplexes
Source: Mol Cell Proteomics. 2017 Dec 8;17(2):216–32. doi: 10.1074/mcp.RA117.000470 (PMC5795388; doi:10.1074/mcp.RA117.000470)
Supplement: Supplemental Data [file supp_RA117.000470_133922_0_supp_23978_fzffwf.zip › spectra_annotation/mito_spectra_annotation/62-1-12-1-37-1.pdf]

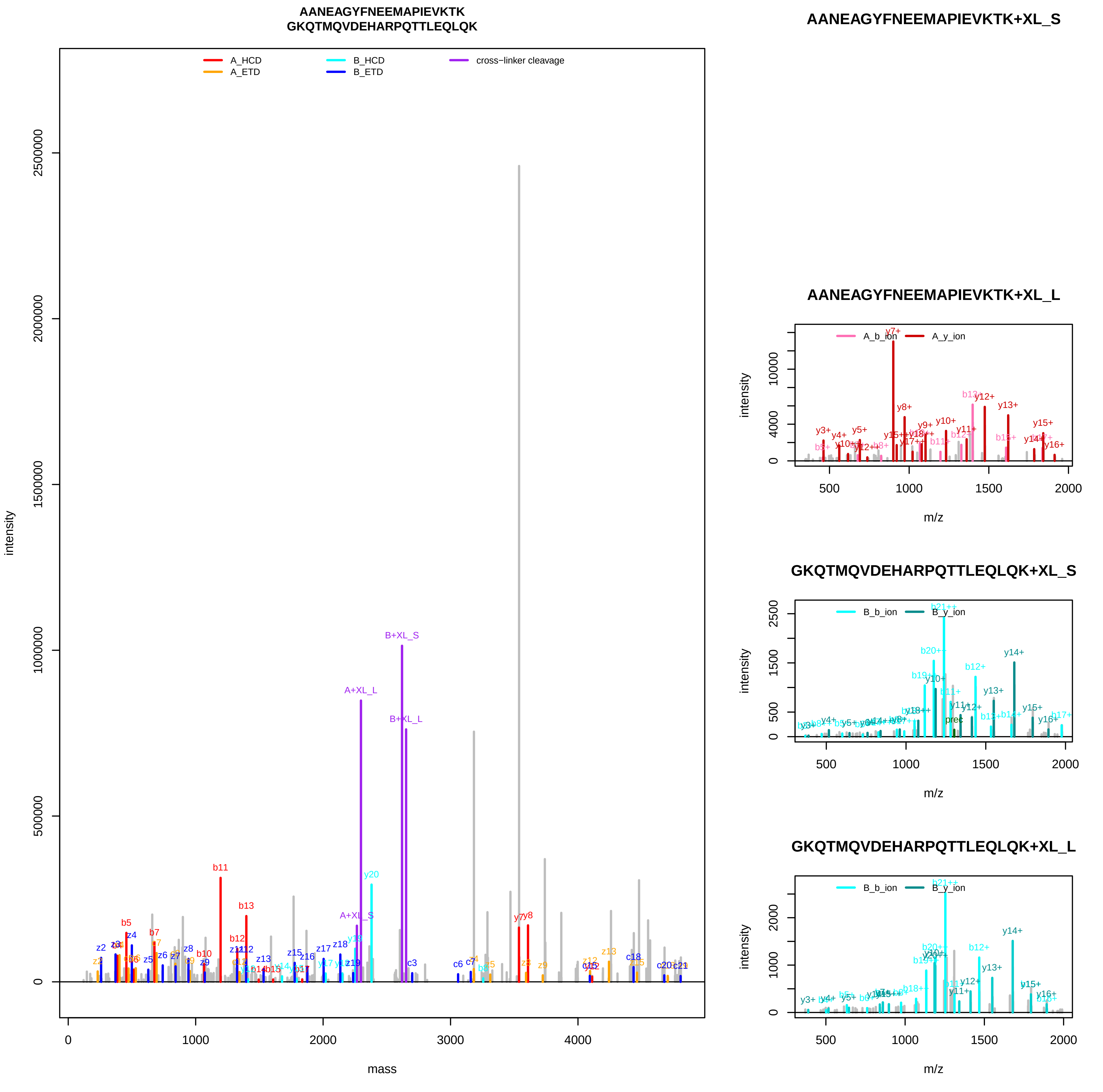

AANEAGYFNEEMAPIEVKTK
GKQTMQVDEHARPQTTLEQLQK
A_HCD
A_ETD
B_HCD
B_ETD
cross−linker cleavage
intensity
mass
0
500000
1000000
1500000
2000000
2500000
0
1000
2000
3000
4000
B+XL_S
A+XL_L
B+XL_L
A+XL_S
b11
b13
b5
b7
b10
b12
y20
y7
y8
z13
c18
c20
c21
c3
c6
c7
AANEAGYFNEEMAPIEVKTK+XL_S
AANEAGYFNEEMAPIEVKTK+XL_L
A_b_ion
A_y_ion
intensity
m/z
0
4000
10000
500
1000
1500
2000
y7+
y8+
y12+
y13+
y15+
y16+
GKQTMQVDEHARPQTTLEQLQK+XL_S
B_b_ion
B_y_ion
intensity
m/z
0
500
1500
2500
500
1000
1500
2000
b21++
b20++
b12+
y14+
y13+
y15+
y16+
GKQTMQVDEHARPQTTLEQLQK+XL_L
B_b_ion
B_y_ion
intensity
m/z
0
1000
2000
500
1000
1500
2000
b21++
y14+
b12+
y13+
y15+
y16+
